# Hydrogen Production by Sorption Enhanced Steam Reforming (SESR) of Biomass in a Fluidised-Bed Reactor Using Combined Multifunctional Particles

**DOI:** 10.3390/ma11050859

**Published:** 2018-05-21

**Authors:** Peter T. Clough, Matthew E. Boot-Handford, Liya Zheng, Zili Zhang, Paul S. Fennell

**Affiliations:** 1 Chemical and Process Engineering Department, Imperial College London, South Kensington, London SW7 2AZ, UK; lyzheng@alum.imr.ac.cn (L.Z.); zili.zhang@imperial.ac.uk (Z.Z.); p.fennell@imperial.ac.uk (P.S.F.); 2 Energy and Power Engineering, Cranfield University, Cranfield, Bedfordshire MK43 0AL, UK

**Keywords:** hydrogen, sorption enhanced steam reforming, combined multifunctional particles, fluidised bed reactor, biomass

## Abstract

The performance of combined CO_2_-sorbent/catalyst particles for sorption enhanced steam reforming (SESR), prepared via a simple mechanical mixing protocol, was studied using a spout-fluidised bed reactor capable of continuous solid fuel (biomass) feeding. The influence of particle size (300–500 and 710–1000 µm), CaO loading (60–100 wt %), Ni-loading (10–40 wt %) and presence of dicalcium silicate support (22.6 wt %) on SESR process performance were investigated. The combined particles were characterised by their density, porosity and CO_2_ carrying capacity with the analysis by thermogravimetric analysis (TGA), Brunauer-Emmett-Teller (BET), Barrett-Joyner-Halenda (BJH) and mercury intrusion porosimetry (MIP). All experiments were conducted with continuous oak biomass feeding at a rate of 0.9 g/min ± 10%, and the reactor was operated at 660 ± 5 °C, 1 atm and 20 ± 2 vol % steam which corresponds to a steam-to-carbon ratio of 1.2:1. Unsupported combined particles containing 21.0 wt % Ni and 79 wt % CaO were the best performing sorbent/catalyst particle screened in this study, when accounting for the cost of Ni and the improvement in H_2_ produced by high Ni content particles. SESR tests with these combined particles produced 61 mmol H_2_/g_biomass_ (122 g H_2_/kg_biomass_) at a purity of 61 vol %. Significant coke formation within the feeding tube and on the surfaces of the particles was observed which was attributed to the low steam to carbon ratio utilised.

## 1. Introduction

Global and national policies, including the Paris agreement [1] and the UK’s Climate Change Act [2], demand that all industries and sectors must decarbonise their emissions of greenhouse gases in order to mitigate the worst effects of climate change. The production of H_2_ is included in these decarbonisation targets, however, most commercially available H_2_ is produced via the steam methane reforming (SMR) process which emits more than seven times as much CO_2_ as H_2_ produced [3]. The integration of carbon capture, utilisation and storage (CCUS) technology is therefore needed to decarbonise hydrogen production [4].

H_2_ is an important chemical feedstock most notably used in the production of ammonia and in the oil refining industry. It is also a versatile, zero-carbon energy vector that has applications as a fuel for both centralised and distributed electricity generation, domestic and industrial heating and transportation [5]; H_2_ could also provide a zero-carbon substitute in existing natural gas networks for domestic and industrial demands [6,7].

Sorption Enhanced Steam Reforming (SESR) is a pre-combustion CO_2_ capture process in which hydrocarbon fuel is reacted with steam in the presence of a CO_2_ sorbent and reforming catalyst to produce decarbonised, high purity H_2_ (Equation (1)) and concentrated CO_2_ suitable for transport and geological storage (or reuse). Assuming a stoichiometric ratio of each reactant, the SESR process overall is mildly exothermic due to the exothermic water-gas shift and carbonation reactions balancing the endothermic steam methane reforming. SESR utilises two reactors: a joint carbonator/gasifier/reformer where CO_2_ is absorbed and H_2_ generated; and a calciner where the sorbent is regenerated, releasing CO_2_; the nickel catalyst may also become partially oxidised [8]. The presence of a CO_2_-sorbent in the reforming stage shifts the equilibrium position of the reactions, in favour of H_2_ production, as described by Le Chatelier’s principle [9].

Equation (1). Simplified, overall SESR reaction:

(1) $${\mathrm{CH}}_{4}+2H_{2}O+\mathrm{CaO}⇌4H_{2}+{\mathrm{CaCO}}_{3}\;\Delta H_{r}{}^{\circ}\;=\;-13.8\;\mathrm{kJ}/\mathrm{mol}$$

CaO is the most commonly studied CO_2_ sorbent for SESR applications due to: (i) sufficiently fast sorption kinetics at a temperature range (500–700 °C) compatible with gasification, reforming and water-gas-shift reactions; (ii) its high CO_2_ carrying capacity; (iii) wide availability and (iv) low cost. Other sorbents investigated for SESR applications include hydrotalcites, dolomites and MgO-based sorbents [10,11,12,13,14,15].

The main drawback with Ca-based sorbents is their widely documented decay in CO_2_ carrying capacity over multiple carbonation/calcination cycles caused by thermal and reactive sintering, attrition and sulphation [16,17]. A substantial research effort has been directed at developing and testing strategies for overcoming the degradation in the cyclic CO_2_ carrying capacity of CaO with varying degrees of success [18,19].

**Figure 1:**
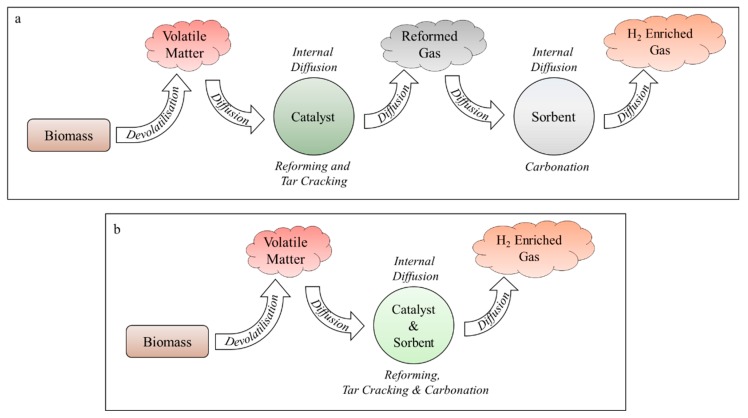
Representation of the overall Sorption Enhanced Steam Reforming (SESR) mechanism and associated mass transfer, diffusional resistances present in during SESR of biomass for separated (**a**) and single particle (**b**) systems.

The reforming/carbonation stage of the SESR process is limited by mass transfer, in particular diffusion of the intermediary and final products between the catalytic and sorption sites [20]. The amalgamation of catalyst and sorbent materials into a single, multifunctional particle is therefore of great interest within the research community [21,22,23,24,25]. The single, combined particle system greatly minimises the effective diffusion distance and prevents dilution of intermediates by the bulk gas, thereby promoting improved mass transfer rates and conversion efficiencies. A schematic showing the effective diffusion pathways for separated- and single-particle systems when biomass is utilised as the feedstock is shown in Figure 1. The best performing combined particle systems reported in the literature are formed of homogeneously mixed catalytic and sorption material [26,27,28].

A wide range of methods for producing combined, multifunctional particles have been reported, from highly-engineered sol-gel [29] and co-precipitation techniques [30,31], to low-engineered techniques such as wet-impregnation [32,33] and wet/physical mixing [30,33,34,35,36]. The highly-engineered methods often require costly chemical precursor reagents and have multiple time-consuming manufacturing steps. On the other hand, low-engineered methods are generally simpler, cheaper and quicker to produce. There is the argument that highly-engineered methods can result in better performing materials. However, the feasibility and cost of scaling up production of these materials and the implications this has on the OPEX of large-scale plants is rarely considered. A further argument is that direct contact between the sorbent/catalyst particles and the fuel (as proposed here) will cause enhanced material degradation due to interactions between ash, sulphur containing gases, coking, and tar deposition. A higher sorbent/catalyst replenishment rate will therefore be needed, thus incentivising the use of a lower cost sorbent/catalyst material.

The use of biomass as the fuel input for SESR is an area of research that is gaining substantial traction since it is a form of negative emissions technology [32,37,38,39]. The use of H_2_ produced by biomass-SESR would therefore result in a net-removal of CO_2_ from the atmosphere. However, difficulties with biomass handling (feeding, conveying, handling and tar production) has meant that the majority of research has been limited to the study of separate gasification and reforming reactors, fixed beds, or thermogravimetric analysers (TGA) with model biomass compounds—all of which are far from representative of an industrial scale operation [13,40,41,42,43,44,45,46,47,48,49,50].

Limited experimental experience is available involving the use of multifunctional sorbent and catalyst combined particles with a biomass SESR process. As such, a spout-fluidised bed reactor was designed and constructed to allow the study of an SESR process with continuous solid fuel feeding. This research aimed to explore the use of combined sorbent and catalyst particles prepared via a simple low-engineered wet mixing process within a biomass SESR process and highlight any operational limitations.

## 2. Materials and Methods

### 2.1. Particle Production

Combined, multifunctional particles were prepared via a wet mixing method. The method was adapted from similar methods described elsewhere in the literature [25,34,51,52,53]. Longcliffe limestone (<90 µm, 98 wt % CaCO_3_, supplied by Longcliffe Quarries Ltd., Matlock, UK) was calcined in a tube furnace in air at 900 °C for 1 h. The produced CaO was allowed to cool under a flow of N_2_ and subsequently transferred to a container and sealed to prevent rehydration and recarbonation. The CaO was then weighed and slowly hydrated by adding deionised (DI) water, initially dropwise, and then in 5 mL aliquots until the required quantity of water was added. In general, approximately 2 mL of water was added for every 1 g of CaO. The amount of water used was in great excess compared to the amount needed to completely hydrate the CaO as the aim was to form a slurry. The water was added slowly to minimise the temperature rise associated with the highly exothermic hydration reaction and prevent boil-off and possible elutriation of the powder from the beaker.

Nickel oxide (NiO, <50 nm (TEM), 99.8 wt % Ni(II)O, supplied by Sigma Aldrich, Gillingham, Dorset, UK ) was then added to the solution and mixed thoroughly by way of a hot-plate with magnetic stirring to achieve a homogenous mixture. The amount of NiO added was calculated to achieve Ni loadings of 10–40 wt % in the final reduced product. The actual Ni-loadings, as confirmed by X-ray Fluorescence (XRF), were 11, 21, 28 and 37 wt %. XRF analysis was performed on all raw materials and the data is available in the supporting information (Table S1).

The hot plate was then set to a temperature of 75 °C, with continuous stirring, and the slurry allowed to thicken into a paste with the consistency needed for extrusion. The paste was then extruded through a selection of sieves with varying mesh apertures. Different sized sieves were chosen to direct the production of different sized particles. The sieves (extrudates still attached) were partially dried in an oven at 110 °C for ~15 min before the extrudates were removed from the sieves. The particles were returned to the oven at the same temperature and allowed to dry completely overnight.

Prior to use, the particles were calcined in a Lenton (Lenton Furnaces, Hope, Hope Valley, UK) horizontal tube furnace at 950 °C for 1 h in air (2 L/min). The cooled, calcined particles were then sieved to the desired size fractions and stored in sealed vessels. Two particle size fractions were produced, 300–500 and 710–1000 µm.

A set of particles supported with dicalcium silicate (C_2_S—notation from cement chemistry) were also produced. C_2_S is a polymorphic spacer that has been shown to provide an effective support for CaO-based sorbents which acts to reopen the pore structure during calcium looping cycling [54,55]. The method for producing C_2_S supported combined catalyst/sorbent particles was adapted from a previously reported method used for producing C_2_S supported CaO [54,55].

Tetraethyl orthosilicate (TEOS, >99%, Sigma Aldrich, St. Gillingham, Dorset, UK) was first hydrolysed in DI water with the aid of HNO_3_, added in sufficient quantity to achieve a pH ≈ 3. This solution was then used to hydrate the CaO. All additional production steps were the same as those described above for the unsupported particles. Only C_2_S supported particles of size fraction 300–500 µm were produced. The C_2_S was formed during the final calcination step in the tube furnace. Particles were prepared with a target Ca:Si molar ratio of 9:1, as recommended by Zhao et al. [55] The actual molar ratio of the final particles was 8.7:1.3 as confirmed by XRD analysis. A table of all materials utilised and sources is presented, Table 1.

The unsupported particles (Table 2) were named according to the quantity of nickel in the particles, i.e., a sample consisting of 21 wt % Ni and 79 wt % CaO was named “21 wt % Ni”. The supported samples were only prepared with a single Ni loading of 21 wt % and therefore named: “C_2_S—21 wt % Ni”. For comparison, separate sorbent (0 wt % Ni) and catalyst (0 wt % CaO) particles were also prepared; these samples were named “CaO only” and “NiO only” respectively.

### 2.2. Particle Characterisation

Particles were physically characterised post calcination. N_2_-sorption analysis (Micrometrics Tristar 3000, Lincoln, Lincolnshire, UK) was carried out to determine the BET surface area and BJH pore size distributions for porous region (5–200 nm). Helium adsorption (Accupyc, Micromeritics—AccuPyc II 1340 Series Pycnometer, Lincoln, Lincolnshire, UK) analysis was used to measure the skeletal densities of the particles. Mercury intrusion porosimetry (MIP, Micromeritrics—AutoPore IV, Lincoln, Lincolnshire, UK) provided the pore size distribution and volume for larger pores up to 100 µm. An arbitrary upper limit of 100 µm was selected, above which the measured pore volume was assumed to be dominated by interstitial particle space rather than true internal pore volume. MIP provides limited information about porosity made up of pore diameters below ~10 nm due to the stresses exerted on the materials by compression of the mercury that can result in particle fracture and breakup. As such, N_2_-adsorption measurements and BJH analysis were favoured when commenting on porosity comprised of pore diameters less than 150 nm [56,57].

XRF (PANanalytical—Epsilon 3*^x^*, Royston, Hertfordshire, UK) was utilised to evaluate the elemental compositions and XRD (CuK*α* radiation, PANalytical Almelo, Royston, Hertfordshire, UK) in combination with GSASII [58] (for rietveld refinement) were used for semi-quantitative analysis phase compositions. FactSage [59] and Thermovader [60] were applied to predict the thermodynamic equilibrium phase compositions of the samples (available in the supporting information, Figure S1).

A TGA (Q5000IR, TA Instruments, Elstree, Hertfordshire, UK) was used to screen the particles prior to testing in the spout-fluidised bed reactor to assess the cyclic CO_2_ carrying capacity under realistic conditions described in detail elsewhere [54]. Briefly, the conditions were carbonation at 650 °C for 5 min under 15 vol % CO_2_ (balanced N_2_) and calcination at 950 °C for 1 min under 100 vol % CO_2_. The CO_2_ concentration was increased proportionally to the sample temperature to prevent unintended over-carbonation or pre-calcination when the temperature was increased from the carbonation to the calcination temperature. A TGA-MS (thermogravimetric analysis - mass spectrometry, Netzsch—TG 209 F1 with QMS 403 D, Selb, Bavaria, Germany) was utilised for analysing the coking on the particles.

## 3. Reactor Description

### 3.1. Reactor and Equipment Design

**Figure 2:**
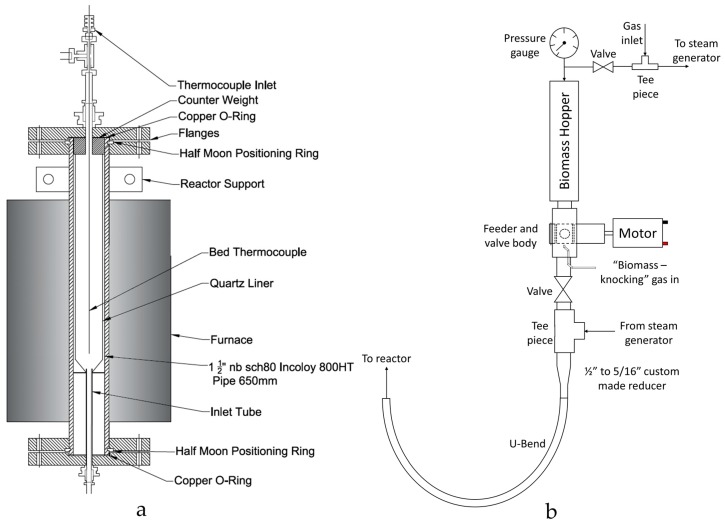
(**a**) Computer Aided Design (CAD) drawing of the reactor body design and details of the major components; (**b**) Solids feeding design for continuous operation.

A new reactor was designed and constructed for this study. Schematic Computer Aided Design (CAD) drawings of the reactor body and continuously operated solids feeding system are displayed in Figure 2. The reactor was based on a spout-fluidised bed design, similar that reported by Zhang et al. [61] This configuration was chosen due to its effective heat transfer and gas-solid mixing. The absence of a gas distributor also allowed for the continuous transfer of solids into the bed entrained by the inlet gas flow. 

### 3.2. Feeding System

The feeding system comprised of a rotating valve arrangement that transported biomass from a hopper into a gas stream which conveyed the biomass into the reactor (Figure 2b). The custom-made plastic (Delrin, Polyoxymethylene supplied by DuPont, Stevenage, Hertfordshire, UK) rotary cup feeder—consisting of four 5 mm holes, each 4 mm deep—was housed inside a Swagelok ½″ plug valve and rotated by a small 6 V DC motor. When biomass feeding was required, the valves above and below the feeding system were opened, and the motor and “biomass-knocking” gas flow were started. The biomass-knocking gas flow refers to a small flow of N_2_ (5 cm^3^/s controlled by a Brooks 5850EM mass flow controller, Veenendaal, The Netherlands) which entered the system beneath the outlet of the feeder and ‘knocked’ the biomass out of the cups. The small flow of gas also helped prevent steam from condensing on the surfaces of the feeding system below the feeding valve which could not be heated due thermal constraints of the valve construct.

The biomass was fed into a ½″ to 5/16″ custom-made adapter- a critical component of the feeding system. The adaptor was designed to provide a smooth, gradual and “ridge-free” transition from the ½″ feeder valve to the 5/16″ tube of the reactor inlet minimising the chance of blockage formation. The biomass and biomass-knocking gas was blended with the rest of the inlet gas flow at the exit of the custom-made adaptor. At this stage, the gas flowrate was above the biomass’ terminal velocity and so the biomass was pneumatically conveyed into the reactor via the U-bend.

### 3.3. Steam Generation System

Steam was produced using a custom-made bubbler and evaporator/distributor column. The system consisted of a 300 mm long 1″ tube, half filled with DI water, wrapped in heating tape to produce a bubbler. N_2_ carrier gas entered from the bottom of the bubbler via a 1/8″ tube with several holes drilled into its length. The gas bubbled through the heated water and in doing so collected water vapour which then passed into a 3 m long distribution coil made of ¼″ tubing and then into a second heated column (1″ tube, 300 mm in length) filled with stainless steel balls (4 mm diameter) to disrupt flow patterns and ensure complete mixing. The entire steam generation system and all downstream gas lines were trace heated. The gas line temperatures were controlled by external type K thermocouples (TC direct), apart from the bubbler which had an internal thermocouple. A water inventory of 75 mL (≈50% of capacity) was maintained within the bubbler using a HPLC pump. The relative standard deviation in steam concentration when utilising this steam generator was ±10% of the set point.

The steam concentration was controlled via the bubbler temperature and carrier gas flow rate set points. The steam concentration at the inlet and outlet of the reactor were monitored via two humidity probes (HMT334, 0–100%, Vaisala, Bury St Edmunds, Suffolk, UK). The steam exiting the reactor was collected in an ice-cooled water and tar trap. A packed bed of CaCl_2_ downstream of the water and tar trap removed residual water vapour from the gas stream prior to the gas analysers.

### 3.4. Reactor Body

The main reactor body was a 650 mm length of Incoloy 800-HT pipe, with an outer diameter of 48.3 mm and a wall thickness of 5.1 mm (nominal pipe size 1½″, schedule 80, Philip Cornes & Co. Ltd., Birmingham, West Midlands, UK). Both ends of the tube were sealed with flanges, half-moon positioning rings and copper O-rings. The bottom flange had a bored through 5/16″ fitting for connecting the support plate/inlet tube (Figure 2) which fits inside the reactor. The reactor main body housed an internal quartz reactor liner (Soham Scientific, Ely, Cambridgeshire, UK) to contain the fluidised bed. The internal quartz reactor consisted of a 33 mm I.D. (1.5 mm wall thickness) quartz tube that tapered in at the base (16 mm I.D.). The hole at the base was designed to sit over the spout jet which provides the inlet for both the gas and biomass. The quartz reactor was sealed against the base plate supporting ring (from which the spout protrudes) using a high temperature Kaowool sealing material. A counterweight positioned on top of the reactor provided the force needed for sealing.

The reactor was externally heated by a 1.5 kW vertical split tube furnace (Lenton Furnaces, Hope, Hope Valley, UK, CSC 12/90/300 V) which was controlled to the set-point temperature using a type N thermocouple attached to the outer wall of the reactor. Type K thermocouples were also positioned in the bed (*via* a Spectite fitting with Viton sealant) and on the outer wall of the reactor, with temperatures logged. Superwool insulating blanket (RS Components Ltd., Corby, Northamptonshire, UK) was used to insulate the reactor not covered by the furnace and all trace heated lines.

### 3.5. Downstream Gas Analysis

The concentrations of CO_2_, CO and CH_4_ in the gas stream exiting the reactor were measured using online non-dispersive infrared analysers (ADC MGA 3000, 0–50 vol % ±1% of the measured value, Hoddesdon, Hertfordshire, UK). O_2_ was measured using a chemical oxygen sensor cell (housed in the same unit). The concentration of H_2_ was measured via a calibrated thermal conductivity detector (ABB EL3020-Caldos27, 0–30 vol % ±1% of span, St. Neots, Cambridgeshire, UK). Post gas analysis the gases were sent to a flare to combust flammable or harmful gases before venting through local ventilation extraction.

### 3.6. Gas Supply

Gas was supplied from cylinders of (i) N_2_ (99.998 vol %, oxygen-free, BOC, Guildford, UK); (ii) CO_2_ (99.9 vol %), (iii) 10 mol. % H_2_ (balance N_2_, BOC) and (iv) air (99.9 vol %, BOC). A single point calibration was performed before each of the SESR experiments using a certified calibration (30 vol % CO_2_, 10 vol % CO, 10 vol % CH_4_, and 10 vol % H_2_, BOC) and the 10 vol % H_2_ gas mixture. Gas flow rates were controlled via calibrated rotameters (Roxspur Measurement & Control Ltd., Sheffield, UK). A summary of the flow rates utilised during each cycle phase is provided in Table 3. During the main SESR phase the *U*/*U*_mf_ was 3.6 for sand, (*ρ*_sand_ = 2600 kg/m^3^, 425–500 µm), calculated using correlation reported by Wen & Yu [62].

### 3.7. Standard Operating Procedure

On completion of a pressure/leak test at 3 bar, the furnace was switched on and the reactor heated up, with a flow of 30 cm^3^/s N_2_ added. The combined sorbent/catalyst particles were dropped in to the bed along with 40 g of silica sand (425–500 µm, *ρ*_sand_ = 2600 kg/m^3^, purity > 98%, David Ball Co., plc, Bourn, Cambridgeshire, UK) through the top of the reactor when the internal reactor temperature had reached 500 °C. Further heating of the bed to the operating temperature (660 °C) resulted in the calcination of any carbonates or hydrates that may have formed during storage, weighing or transfer.

The NiO component of the sorbent/catalyst particle was then reduced in 5 vol % H_2_ (balanced N_2_) at 650 °C for 30 min. Prior TPR analysis as well as online measurements of the steam and H_2_ concentration at the outlet of the reactor confirmed that reduction of the Ni catalyst reached completion under these conditions. Following the reduction step, the gas flow was switched to 100 vol % N_2_ to purge the system of H_2_, then the steam generator heating and gas flow switched on.

Once a stable steam concentration was achieved, the biomass feeding system was turned on and the SESR reactions initiated. The SESR experiments were conducted at 660 ± 5 °C, 1 atm and 20 ± 2 vol % steam, which produced a stoichiometric molar steam to carbon ratio of 1.2:1. Oak wood (212–300 µm) was used as the biomass in this work and fed at a rate of 0.9 g/min ±10% relative standard deviation. Proximate and ultimate analysis, and heating value of the biomass (estimated by its ultimate analysis data) is available in Table 4.

After a set 3 min time period (determined during the reactor commissioning and experimental design phase), the biomass feeding was turned off and the main N_2_ gas flow set to bypass the steam generation system, effectively shutting off the steam flow to the reactor. The reactor was then heated to 750 °C and the CaCO_3_ formed during the SESR process was calcined. On completion of the calcination reaction, air was added to the reactor to combust coke produced during the SESR and any char remaining in the bed. Online data logging of the gas analyser signals, relative humidity probes and thermocouples was achieved using a data acquisition card (Redlab 1208FS DAQ card, Meilhaus Computing, supplied by RS Components Ltd., Corby, Northamptonshire, UK) and an Agilent VEE control program.

## 4. Results and Discussion

### 4.1. Particle Characterisation

**Figure 3:**
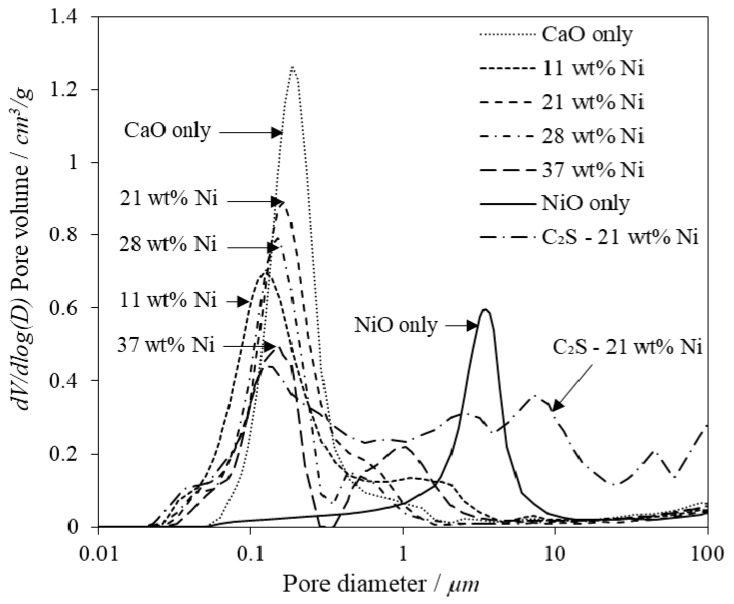
Pore volume distribution, *dV/dlog* (*D*), from Mercury Intrusion Porosimetry (MIP) analysis for combined particles of various compositions.

To assess the impact of nickel oxide addition on the pore structure of the combined particles, MIP, BJH and BET analysis was conducted. The pore size distributions as measured by MIP indicate that all of the materials were highly macro-porous (Figure 3).

Increasing the concentration of Ni loading resulted in the development of a bi-modal pore distribution where the CaO grains formed pores around 0.1 µm and the NiO grains created pores around 1 µm. The increase in Ni content also caused a decrease in the BET surface area and most importantly the surface area and pore volume within the optimal carbonation pore diameter range, 10–100 nm (Table 5) [56,64]. The C_2_S supported sample had a much broader pore distribution compared to the equivalent unsupported 21 wt % Ni combined particle, indicating that the C_2_S support prevented grain agglomeration and facilitated a higher pore volume, surface area and porosity, as shown in Table 5. This aligns well with research by Zhao et al., who showed the addition of 20% C_2_S to a CaO matrix can more than double the BET surface area and pore volume of a CaO matrix [65]. The smaller particle size fraction had a consistently greater pore volume and surface area, likely because of external surface area being the dominant source of total surface area. A slight decrease in porosity with increased Ni content was observed, but no more than ±10% difference was observed which is within experimental error for these analytical techniques.

Thermodynamic analysis of the Ni-CaO-SiO_2_ system at 950 °C, 1 atm, indicated that no interactions between the Ni and SiO_2_ ions were possible upon calcination at 950 °C (phase diagrams provided in supporting information). XRD analysis provided further confirmation. XRD analysis and Rietveld refinement indicated that the C_2_S support loading in the calcined particles was 22.6 wt % although 28.4 wt % was theoretically possible.

**Figure 4:**
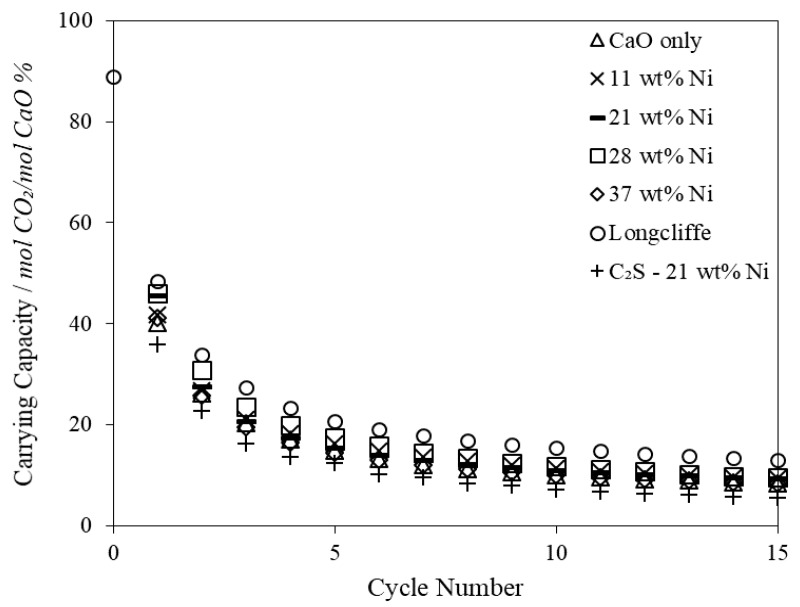
CO_2_ carrying capacity in moles of CO_2_ absorbed per mole of CaO as a percentage for combined particles of various compositions (300–500 µm). Carbonation at 650 °C for 5 min under 15 vol % CO_2_ (balanced N_2_) and calcination at 950 °C for 1 min under 100 vol % CO_2_. The CO_2_ concentration was increased proportionally to the sample temperature to prevent unintended over-carbonation or pre-calcination when the temperature was increased from the carbonation to the calcination temperature.

There was little difference in the CO_2_ carrying capacity (per mol CaO) measured in the TGA over 15 cycles between the CaO only and 37 wt % Ni combined particle despite the much lower surface area and optimal pore volume (10–100 nm) of the 37 wt % Ni sample (Table 5). The degradation characteristics of a raw sample of Longcliffe limestone is also presented in Figure 4, which was cycled under the same severe conditions as the combined particles thereby highlighting the impact of the particle production process. The C_2_S supported combined particles displayed the lowest CO_2_ carrying capacity, likely due to its initially enhanced pore structure enabling CO_2_ enhanced sintering to take place at a greater percentage of the particles CaO sites; this result is in line with other work using this material [54]. Importantly though, the difference between the combined particles with varying Ni contents was small, which indicates that the impact of nickel addition on the sorbents ability to absorb CO_2_ was greatly outweighed by the high-temperature CO_2_ sintering.

### 4.2. Operation in the Spout-Fluidised Bed Reactor

**Figure 5:**
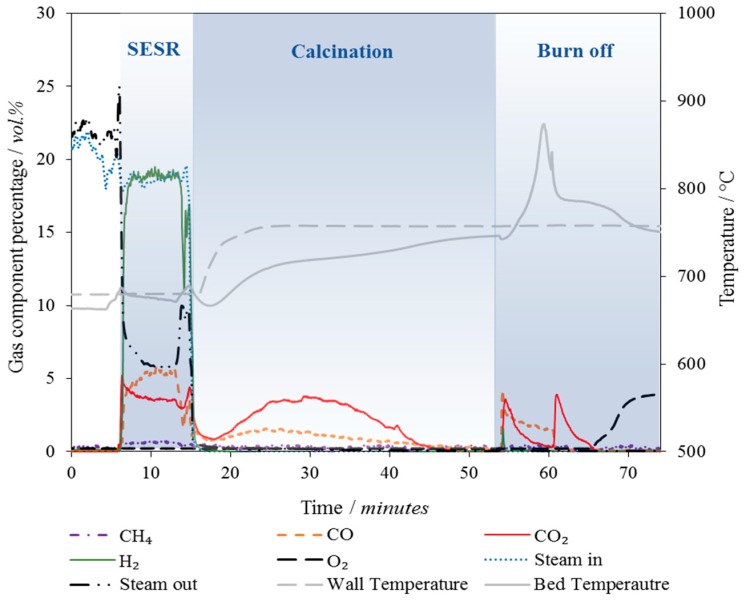
The gas and temperature response for 10 min of biomass Sorption Enhanced Steam Reforming (SESR), followed by calcination and coke burn-off.

A typical outlet gas concentration and temperature profile measured during biomass SESR is displayed in Figure 5, showing the three sequential stages of SESR, calcination and burn-off reactions and the impact on bed temperature and gas production. The SESR experiments were conducted at 660 ± 5 °C, 1 atm and 20 ± 2 vol % steam, which produced a stoichiometric molar steam to carbon ratio of 1.2:1. It is understood that in industrial operation an excess of steam would be used to minimise the potential for coking and to ensure reactions were not limited by the steam concentration [66]. Unfortunately, this was not achievable here, due to the large total flow rate that was required to transport the biomass into the reactor, which impacted on the production capability of the steam generation system. The molar ratio of calcium oxide to carbon (CaO:C) was kept at a constant value of 44. CaO constituted 18 g of the total combined particles sample mass for each experiment; the total amount of combined particles added varied depended on the Ni content. The large excess of CaO (compared to the amount of CO_2_ that could be released) used here should have provided the opportunity to operate the reactor for extended periods of time under steady state conditions.

As shown in Figure 5 (which shows an extended run to demonstrate the steady state period), once the biomass feeding was turned on, at *t* ≈ 5 mins, the H_2_ concentration immediately rose and outlet steam concentration fell; the production of CO, CO_2_ and CH_4_ was also observed. Steady state operation continued for ~10 min after which the build-up of coke within the feeding tube prevented stable biomass feeding. The biomass feeding system was subsequently stopped, steam production turned off and the reactor temperature increased to 750 °C for calcination. The CO measured in the outlet gases during calcination was attributed to the reverse Boudouard reaction (Equation (2)) i.e., the CO_2_ released from calcination reacting with residual char remaining in the bed and coke deposits that had formed on the surfaces of the particles. This was confirmed by thermodynamic modelling with ThermoVader [60], which calculated the theoretical volume fraction of CO to start the heat period (650 °C) at 73 vol % and finish the heat up period (750 °C) at 91 vol %, whereas CO_2_ decreased from 27 vol % to 5 vol % during the heat up period.

Equation (2). Reverse Boudouard:

(2) $$C+{\mathrm{CO}}_{2}⇌2\mathrm{CO}\;\Delta H_{r}{}^{\circ}\;=\;+172\;\mathrm{kJ}/\mathrm{mol}$$

The endothermic calcination reaction acted to retard the rate at which the bed temperature could be increased. As such, the calcination reaction was near completion by the time the bed temperature had reached the set point. Here, calcination was conducted in an N_2_ atmosphere to allow accurate measurement of the CO_2_ (and CO) released during the calcination period which was needed for mass balance calculations.

The average carbonation conversion (determined from the CO_2_ released during calcination) by the unsupported combined particles was 18.2% and 32.8%, for the large and small size fractions respectively. The carbonation conversion by the C_2_S supported particles was even higher at 59.3%, demonstrating the diffusion limitation of the larger particles and the benefit of support addition.

On completion of the calcination period, air was mixed into the inlet to give an O_2_ concentration of ~3.6 vol %. The oxygen concentration was low in order to minimise the rise in bed temperature which could have damaged the reactor. The O_2_ incompletely combusted/gasified the char in the feeding tube followed by the coke on the surfaces on the particles resulting in the two CO_2_ spikes observed in Figure 5. CO was generated during this burn-off period as well due to the incomplete combustion and reverse Boudouard reactions.

A mass balance was conducted around each of these three reaction periods, SESR, calcination and burn-off; and a total mass balance across the whole reaction period was also conducted. All experiments were repeated in triplicate and if the mass balance was more than 30% off, the run was not used in the final data analysis. The average closure of all runs using unsupported particles was 100.4 ± 15.4% for C, H and O, and the average closure of all runs using supported particles was 115.0 ± 10.7% for C, H and O. Some degree of error was inevitable and the two biggest sources of error were the stability of the biomass feeding system and the steam generator. To minimise the error multiple calibrations of both systems were conducted prior to starting the experimental campaign so that all influencing parameters were controlled.

### 4.3. Gas Production during SESR

**Figure 6:**
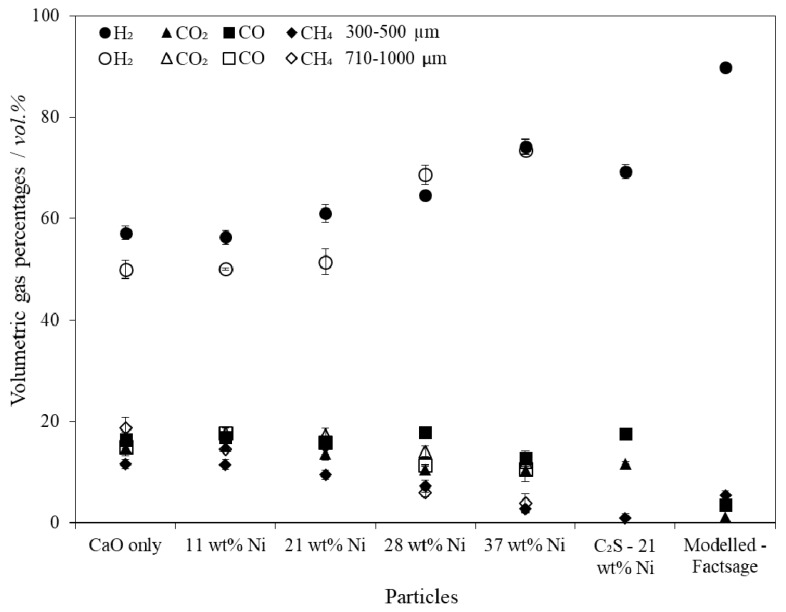
Volumetric gas percentages of the product gas (dry and N_2_ free) averaged over the steady state period during biomass Sorption Enhanced Steam Reforming (SESR) in a fluidised bed reactor with varying compositions of combined particles.

The volumetric percentages of the gas components (H_2_, CO_2_, CO and CH_4_) indicate that in general a higher Ni loading contributed to an increased H_2_ purity and a decrease in the concentration of other gases (Figure 6). There was an observable difference in H_2_ production purity between the smallest and largest particles, which was likely caused by the diffusion of reactant species to/from the active sites. This implies that the reactions occurring were dependent upon the effectiveness factors for the combined particles. This effect diminished at the highest nickel contents which indicates that the diffusional limitations of the larger particles was compensated by the large amount of Ni at the exterior of the pore walls.

The addition of the C_2_S support caused a significant decrease in the production of CH_4_, resulting in a higher H_2_ purity compared to the equivalent unsupported combined particle. The difference in CH_4_ production was attributed to the higher porosity of the C_2_S supported particles (76% compared to 65% for the equivalent unsupported combined particle) and the C_2_S support providing access to more internal area of the particles. The increased particle porosity provided the reactant gases with better access to the Ni sites. It is unlikely that any additional reactions between the C_2_S support and the gas components took place, but this was not confirmed in this study. 

**Figure 7:**
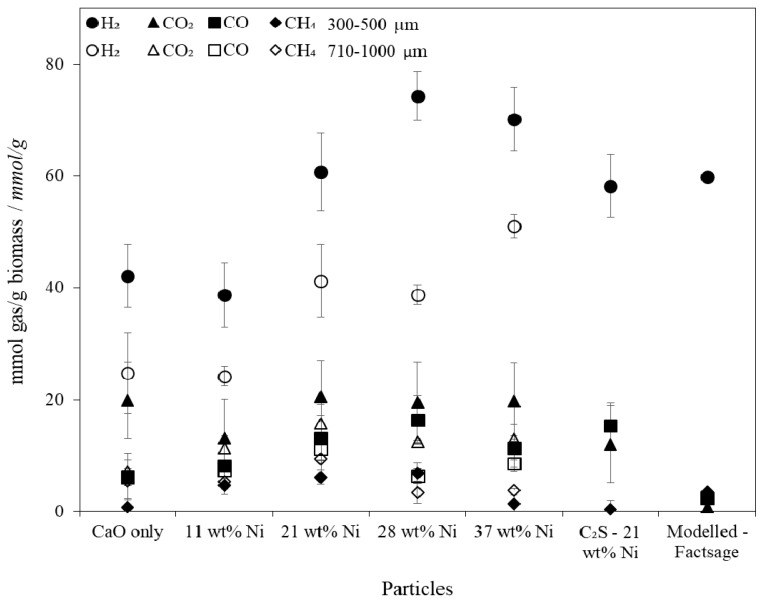
Millimoles of each product gas per gram of biomass from whole reaction period during biomass Sorption Enhanced Steam Reforming (SESR) in a fluidised bed reactor with varying compositions of combined particles.

The gas purities approached the thermodynamic limit for the system (modelled using FactSage [59]) but were limited by intra- and inter-particle mass transfer diffusion (Figure 6). Intra-particle diffusion limitations were brought about by: (i) the initial particle size and morphology; (ii) the gradual coking of the particles’ surfaces, and; (iii) the formation of a CaCO_3_ product layer. Furthermore, the difference between the thermodynamically predicted gas yields and those achieved (Figure 7) was also partially due to the gasification of biomass in the freeboard zone, the elutriation of fine chars, and tar production.

**Figure 8:**
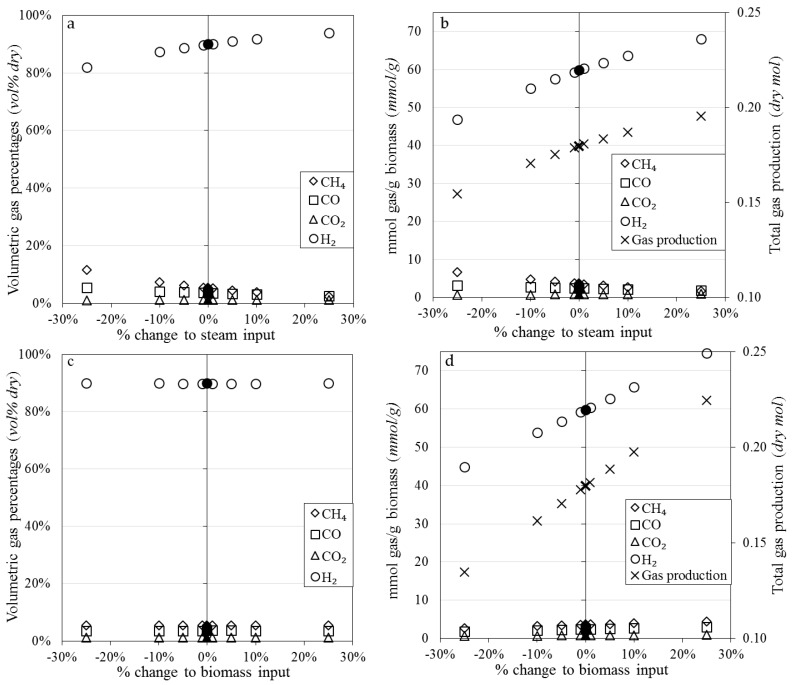
Sensitivity analysis conducted using modelling within FactSage. Figures on the top row (**a**,**b**) show the effect of varying the amount of steam fed into the reactor and the figures on the bottom row (**c**,**d**) indicate the effect varying the amount of biomass fed into the reactor.

The molar yields of each component gas were normalised with respect to the mass of biomass fed into the system to allow comparison with other published work (Figure 7). The gas yields reported in Figure 7 were calculated for the entire SESR reaction period, not just the steady state period (as displayed in Figure 6). As such, the normalised molar gas yields have a much larger associated error and trends between gas yields and nickel loadings are less clear. Online gas concentration measurements were also susceptible to small deviations in the stability of the steam generation and biomass feeding. A sensitivity analysis with respect to steam and biomass input was therefore conducted using FactSage where a 1, 5, 10 and 25% change in the steam and biomass input feeds was simulated (Figure 8). The sensitivity analysis revealed that variations in the steam inlet concentration and biomass feeding rate had a significant effect on the equilibrium position of the reforming and water-gas shift reactions which would in turn affect the measured gas composition and yield. The amount and purity of H_2_ produced during the experimental results could have been produced by varying the biomass and steam inputs by only 10% each, which is within the relative standard deviations measured (±10% for biomass feeding and ± 10% for steam generation) during calibration. To achieve the values of CO_2_, CO and CH_4_ shown in Figure 7, a low utilisation rate of the CaO must have occurred as they were not represented by the sensitivity analysis. It is likely the error ranges and values shown within Figure 7 are a consequence of a combination of factors including the steam and biomass feeding stability and the kinetics of the CO_2_ sorbent.

Ni loadings of up to 21 wt % had little influence on the total gas yields, which was attributed to the shielding of the catalytic sites by the CaO matrix and growing CaCO_3_ product layer. The addition of an extra ~16 wt % Ni in the combined particles (from 21 to 37 wt %) resulted in a small improvement in the H_2_ yield of 10 mmol H_2_ per gram of biomass and an improvement in H_2_ purity of ~10 percentage points; but to the detriment of raw material costs which were almost doubled (Table 6). The experimental data and raw material costs presented here indicates little benefit in increasing the Ni content beyond ~25 wt %, however a more rigorous economic analysis (including the costs of all upstream and downstream processes and fuel/electricity costs) to assess the cost-benefit of additional H_2_ production with increased Ni loading is needed to confirm this conclusion.

**Figure 9:**
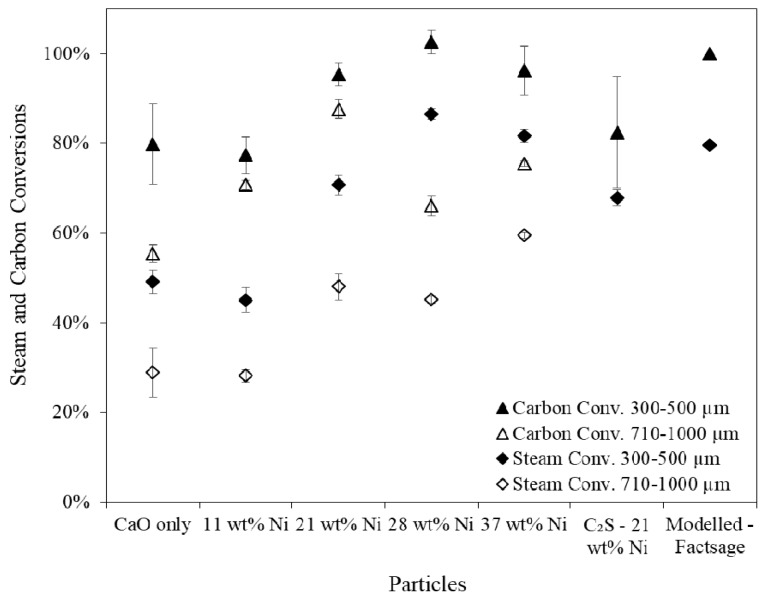
Steam and carbon conversions over the whole Sorption Enhanced Steam Reforming (SESR) reaction period, whereby the carbon conversions include the CO_2_ released during the calcination stage. SESR of biomass in a fluidised bed reactor with varying compositions of combined particles.

Significant differences in the amount of steam reacted was observed when using different particle size fractions (Figure 9). Smaller particles with higher nickel contents were more effective at facilitating the steam reforming and water-gas shift reactions. The influence of Ni loading on the carbon conversion (gasification extent) above a threshold of 21 wt % Ni was limited. The higher carbon conversions observed when using the smaller particles was attributed the greater equilibrium shifting effect caused by the greater availability of the CaO and Ni sites (i.e., higher effectiveness factor). The steam and carbon conversions observed for the C_2_S supported particles similar albeit slightly worse than for the unsupported particles with equal Ni loading. The higher H_2_ purity observed in the case of the C_2_S supported particles was therefore due to an enhancement in the catalytic conversion of methane compared with the unsupported combined particles with equivalent Ni loading.

The unsupported 21 wt % Ni combined particles with a H_2_ purity of 61% and a H_2_ yield of 61 mmol H_2_/g_biomass_ (122 g H_2_/kg_biomass_) have produced results consistent with similar studies reported in the literature. Notably, Arregi et al., who utilised a two stage reactor (spout-fluidised bed for biomass pyrolysis operating at 500 °C, the gases from which were passed into second fluidised bed reactor for SESR, the second reactor was operating at 600 °C and S/C ratio of 7.7 to produce the noted optimal result) and achieved a value of 58 mmol H_2_/g pine wood biomass (117 g H_2_/kg_biomass_) [67]. Further to this, Ji et al. recently reported H_2_ yields of 25 mmol H_2_/g_biomass_ (50 g H_2_/kg_biomass_) for SER of biomass using C_2_S supported combined particles a TGA-MS over a wide range of temperatures. Unfortunately, a direct comparison is not possible since the authors did not use steam in their tests, but this comparison does go some way in illustrating the substantial improvement—in terms of providing a better approximation of industrial SER environment (gas-solid mixing, steam input, solid-solid mechanical interactions)—that can be made by using a fluidised bed reactor system as was used here [38].

### 4.4. Coke Formation on Particles

Severe coke deposition on the combined particles was an understandable consequence of the low steam to carbon ratios utilised. The selectivity for coke formation and deposition was calculated and the results of which are displayed in Table 7. Coke selectivity was calculated as the ratio of carbon burnt off during the burn-off stage and the amount of carbon converted to CO_2_, CO and CH_4_ in the SESR phase. The preference for coke deposition when utilising the largest particle size is highlighted by reference to the smallest particle size and this difference was likely caused by product layer formation and a greater availability of Ni and CaO sites for tar cracking. The effect of nickel dispersion within catalysts has been studied recently and this research has demonstrated that interactions between the support material and the Ni catalyst are key to reducing coking and increasing sintering resistance [68,69]. In general, it appears that the addition of nickel to the overall particle matrix made little difference on the cracking/degradation of the coke.

**Figure 10:**
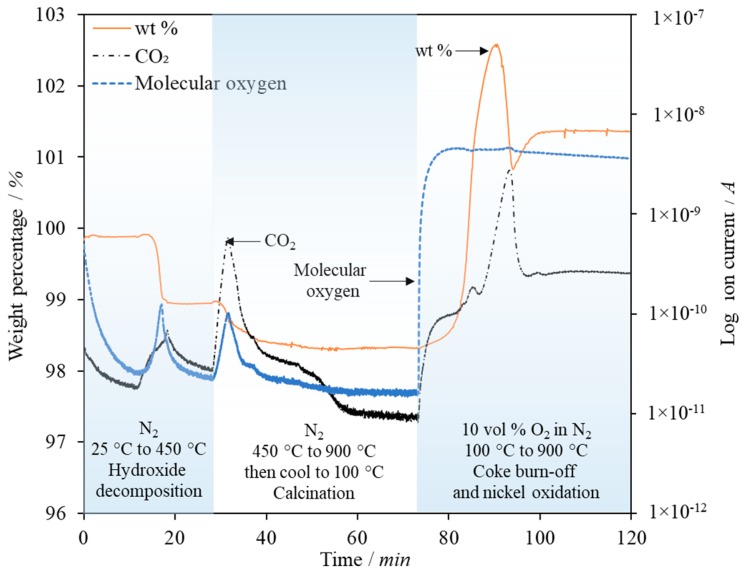
Thermogravimetric Analysis (TGA) weight change as a percentage of the initial mass and mass spectrometer response for CO_2_ and molecular oxygen for a sample of coked combined particles composed of CaO and Ni (21 wt % Ni), post Sorption Enhanced Steam Reforming (SESR) but prior to calcination and burn off.

To investigate the coke deposition further, a sample of the 21 wt % Ni combined particles were recovered from the reactor post-SESR but prior to calcination and burn-off was analysed by Temperature Programmed Oxidation (TPO) in a TGA-MS (Figure 10). The sample was first heated under N_2_ to 900 °C at 10 °C/min to decompose any hydrates or carbonates that had formed during the SESR and cooling down processes. The sample was then cooled to 100 °C at which point the gas inlet was switched to air. The temperature was then ramped at 10 °C/min to 900 °C again for TPO analysis. Determination of the temperature at which the coke decomposes provides information pertaining to the type of coke present [70]. Four main types of coke exist: surface carbides, amorphous films/filaments, graphitic films and graphitic whiskers/filaments. In general, the more graphitic the coke, the more difficult it is to decompose and the more potentially detrimental to the particles it is—graphitic carbon will not only block the active sites but the formation and growth of graphitic coke whiskers can lead to particle breakage [71].

**Figure 11:**
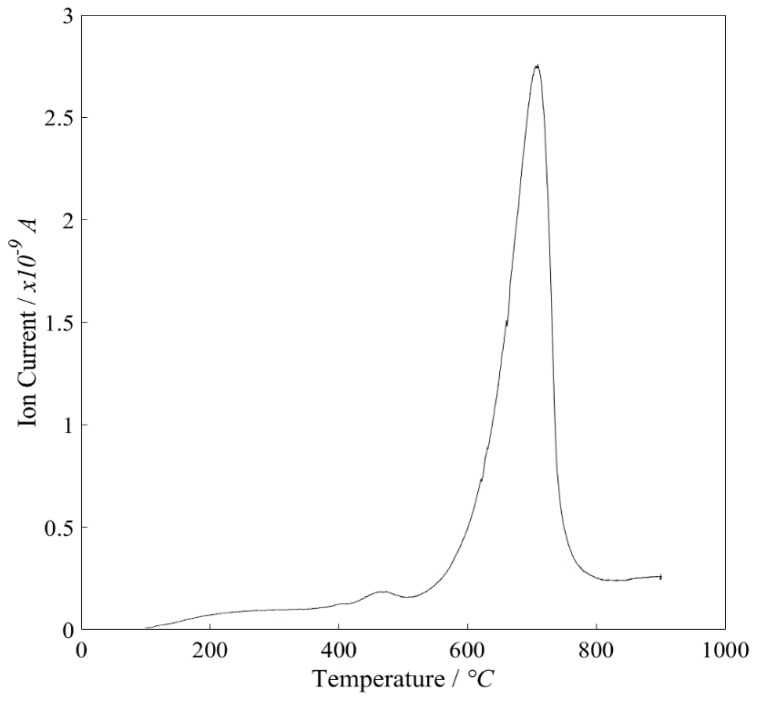
Temperature programmed oxidation showing the CO_2_ mass spectrometry trace against temperature of the Thermogravimetric Analysis (TGA) for a sample of coked combined particles composed of CaO and Ni (21 wt % Ni) post Sorption Enhanced Steam Reforming (SESR) but prior to calcination and burn off.

Most of the coke decomposed between 600 and 800 °C, indicating that the coke was likely of graphitic nature, although it should be noted that the TGA calcination pre-treatment step could have altered the nature of the coke (Figure 11). Without TEM/SEM/Raman spectroscopy (outside of this research) it is difficult to assess accurately the type of coke formed and the analysis here is a best-guess without the availability of further data. Recently, the incorporation of iron into a Ni-based catalyst was shown to impede the rate of carbon deposition; the authors attributed the activity of the Fe to the multiple oxidation states of the iron oxide lattice and its ability to simultaneously oxidise coke whilst being oxidised itself by steam [72]. This could certainly be an interesting area to explore in the future of coke reduction, but caution must be taken during the particle manufacturing steps, as some side interactions between Ca and Fe can occur. A higher steam to carbon (biomass) ratio, above those possible in this study, is also known to inhibit carbon formation [73].

## 5. Conclusions

Sorption enhanced steam reforming (SESR) of biomass using dual-function catalyst/CO_2_ sorbent combined particles was studied using a specially constructed single-stage spout-fluidised bed reactor, capable of continuous solid fuel feeding.

A set of combined particles of varying with varying CaO to Ni ratios were produced by a low-engineered wet mechanical mixing method. The influence of incorporating a dicalcium silicate support was also studied. Two particle size fractions were produced and their material properties characterised.

SESR performance testing in the fluidised spout-bed reactor revealed that the larger particle size fraction suffered from internal diffusional resistance which limited the extent of the SESR reactions. The unsupported particles with 21 wt % Ni produced 61 mmol H_2_/g_biomass_ (122 g H_2_/kg_biomass_) at a purity of 61 vol % at 660 °C, 1 atm and a steam to carbon ratio of 1.2:1, which proved to be the most optimal combined particle when accounting for the raw material costs and the increased H_2_ yield and purity achieved with greater Ni contents. The H_2_ yields and purities achieved are consistent with the best reported results in the literature.

Coking on the surfaces of the particles and within the reactors’ feeding tube limited the long-term operation of the reactor but further development of the steam generation system to allow for higher steam to carbon ratios is expected to alleviate this issue. Further work should investigate the use of these combined particles over multiple cycles in a spouting bed reactor with higher steam to carbon ratios.

## Figures and Tables

**Table 1:** Summary of materials utilised.

Materials	Form	Purity	Source
Longcliffe limestone	<90 µm	98 wt % CaCO_3_	Longcliffe Quarries Ltd., UK
Nickel oxide	<50 nm	99.8 wt %	Sigma Aldrich
Tetraethyl orthosilicate (TEOS)	Liquid	>99%	Sigma Aldrich
Deionised (DI) water	Liquid	>99%	Produced on site

**Table 2:** Summary of combined particles produced.

Sample Name	CaO Content/wt %	Ni Content/wt %	C_2_S Content/wt %	Impurities/wt %
CaO only	98.0			2.0
11 wt % Ni	87.2	10.9		1.8
21 wt % Ni	77.4	20.6		1.6
28 wt % Ni	70.6	28.1		1.4
37 wt % Ni	61.7	36.6		1.3
NiO only		99.8		0.2
C_2_S—21 wt % Ni	50.8	21	22.6	5.6

**Table 3:** Calibrated flow rates utilised during each cycle phase.

Cycle Phase	Gas	Flow Rate (cm^3^/s)
H_2_ reduction	N_2_—Main	30
	10 vol % H_2_ in N_2_	30
SESR	N_2_—Main	59
	N_2_—“Biomass-knocking”	5
	Steam	16
Calcination	N_2_	59
Burn off	N_2_	50
	Air	10

**Table 4:** Proximate and ultimate analysis of the oak wood biomass. Higher heating value (HHV) estimated using work by Sheng and Azevedo [63]. “-” indicates that the concentration was below the level of detection.

Proximate Analysis (wt %)				Ultimate Analysis (wt %)					HHV (MJ/kg)
Moisture Content	Volatile Matter	Fixed Carbon	Ash	C	H	N	S	O	
1.3	80.0	14.2	4.5	45.7	6.1	-	-	48.3	18.8

**Table 5:** Combined, catalytic and sorbent containing particles (300–500 and 710–1000 µm) characteristics from Brunauer-Emmett-Teller (BET), Barrett-Joyner-Halenda (BJH) and mercury intrusion porosimetry (MIP) analysis, density and porosity calculated for 300-500 µm combined particles only.

Sample	BET Surface Area (m^2^/g)		Pore Volume in 10–100 nm Range (BJH, cm^3^/g)		Pore Surface Area in 10–100 nm Range (BJH, m^2^/g)		Skeletal Density (g/cm^3^)	Envelope Density (g/cm^3^)	Porosity (%)
	300–500 µm	710–1000 µm	300–500 µm	710–1000 µm	300–500 µm	710–1000 µm			
CaO only	16.07	14.52	0.064	0.058	5.87	5.15	3.31	1.19	64.1
11 wt % Ni	11.32	10.65	0.050	0.048	4.79	4.41	3.23	1.19	63.2
21 wt % Ni	9.47	8.05	0.032	0.027	3.22	2.59	3.63	1.29	64.6
28 wt % Ni	9.16	8.97	0.034	0.033	3.42	3.25	3.72	1.44	61.2
37 wt % Ni	7.10	6.62	0.030	0.028	2.73	2.46	3.93	1.62	58.8
NiO only	0.95	0.93	0.005	0.005	0.45	0.42	5.93	2.25	62.1
C_2_S-21 wt % Ni	9.11	-	0.038	-	4.42	-	3.54	0.86	75.8

**Table 6:** Raw material costs for combined particles. Yields of H_2_ per gram of biomass was estimated based on an average between the largest and smallest particle sizes due to the larger uncertainty of the data. Costs estimated from Alibaba.com, ~35 US$_2017_ per kg NiO (assuming NiO as the Ni source), ~0.2 US$_2017_ per kg CaCO_3_.

wt % Ni	wt % NiO	H_2_ vol % Purity Produced	H_2_ mmol/g_biomass_ Produced	US$_2017_ Cost of NiO Per kg of Combined Particles	US$_2017_ Cost of CaCO_3_ Per Kg of Combined Particles	Total Raw Material Cost (US$_2017_) Per kg of Combined Particles
11	14	58	32	4.90	0.17	≈5
21	26	61	50	9.10	0.15	≈9
28	36	64	55	12.60	0.13	≈13
37	47	72	60	16.50	0.11	≈17

**Table 7:** Coke selectivity for the various combined particles utilised.

Sample	Coke Selectivity (mg C/g C_Converted_)	
	300–500 µm	710–1000 µm
CaO only	0.38	0.98
11 wt % Ni	0.55	1.34
21 wt % Ni	0.55	1.07
28 wt % Ni	0.66	1.02
37 wt % Ni	0.46	1.33
C_2_S—21 wt % Ni	0.52	-
Modelled—Factsage	0	0

## Supplementary Materials

The following are available online at http://www.mdpi.com/1996-1944/11/5/859/s1, Table S1: X-ray Florescence (XRF) data for the raw materials utilised. “-” indicates that the concentration was below the level of detection. ^†^ Calcium content assumed to be as CaO, Figure S1: Phase diagram indicating the thermodynamically stable species that could be formed when CaO, SiO_2_ and NiO are present at 950 °C, produced in FactSage. The red circle indicates the composition of a material representing ~64 wt % CaO, ~10 wt % SiO_2_, ~26 wt % NiO.

## Abbreviations

Δ*H*_r_°	Enthalpy of reaction at 298 K, kJ/mol
*ρ*_sand_	Density of sand, kg/m^3^
*U*	Fluidisation velocity, cm/s
*U*_mf_	Minimum fluidisation velocity, cm/s
