# Supplementary material for: Hydrogen Production by Sorption Enhanced Steam Reforming (SESR) of Biomass in a Fluidised-Bed Reactor Using Combined Multifunctional Particles
Source: Materials (Basel). 2018 May 21;11(5):859. doi: 10.3390/ma11050859 (PMC5978236; doi:10.3390/ma11050859)
Supplement: Supplementary file 1 [file materials-11-00859-s001.pdf]

Supporting Information for:

Hydrogen Production by Sorption Enhanced Steam Reforming (SESR) of Biomass in a Fluidised-Bed Reactor using Combined Multifunctional Particles

Peter T. Clough*, Matthew E. Boot-Handford*, Liya Zheng, Zili Zhang, Paul S. Fennell

Table S1. X-ray Florescence (XRF) data for the raw materials utilised. "-" indicates that the concentration was below the level of detection. † Calcium content assumed to be as CaO.

Species mol% by XRF	Longcliffe (supplied by Longcliffe Quarries Ltd, UK)	NiO (Supplied by Sigma Aldrich)
$CaCO_3$	98.89	0.07†
NiO	-	99.70
SiO_2	0.16	0.08
Fe_2O_3	0.01	-
Al_2O_3	0.10	0.14
MgO	0.50	-
K_2O	0.05	-
MnO	0.29	-
**Totals**	**100.00**	**99.99**

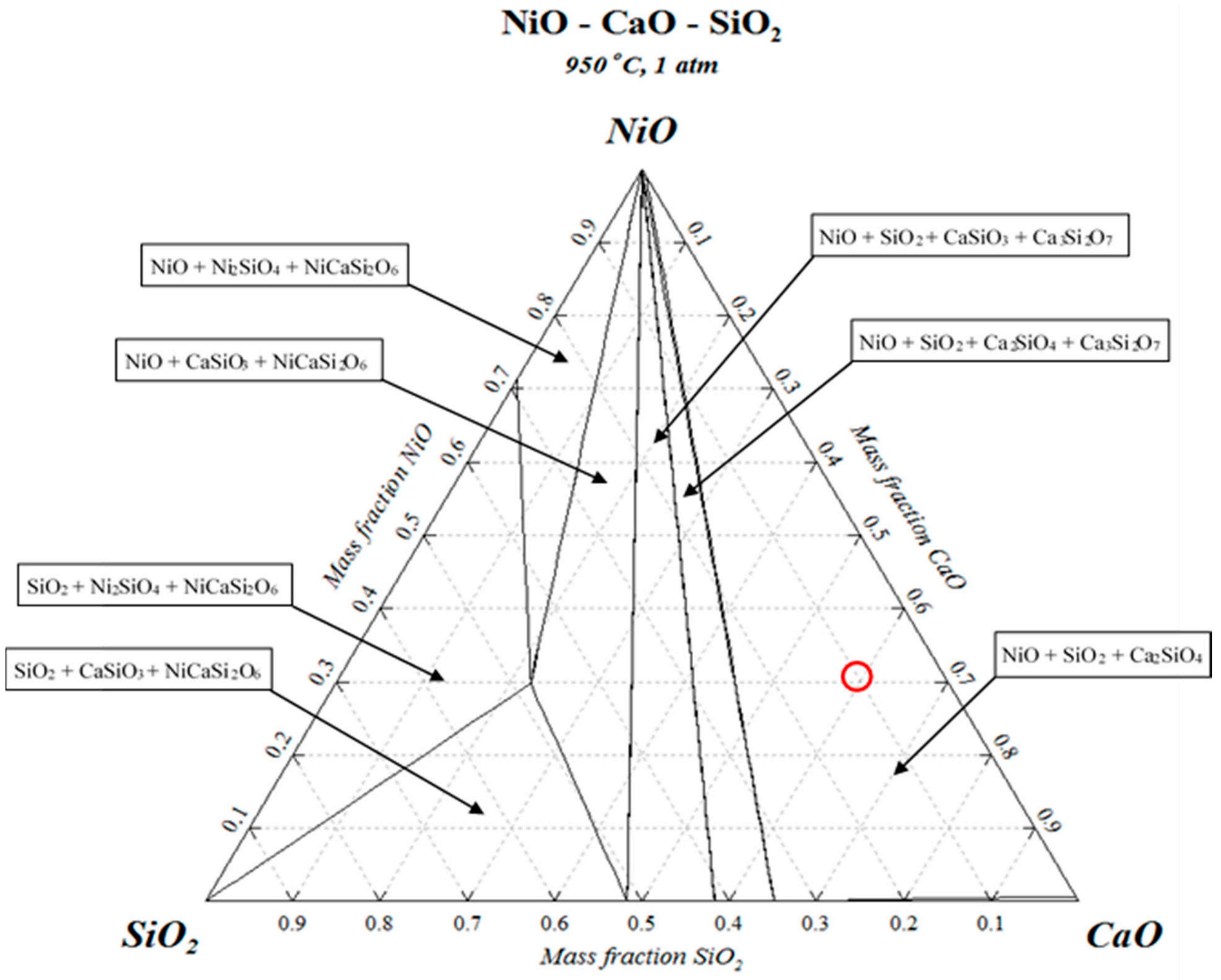

Figure S1. Phase diagram indicating the thermodynamically stable species that could be formed when CaO, SiO_2 and NiO are present at 950 °C, produced in FactSage.[1] The red circle indicates the composition of a material representing ~64 wt.% CaO, ~10 wt.% SiO_2, ~26 wt.% NiO.

References

1. Bale, C. W.; Bélisle, E.; Chartrand, P.; Decterov, S. A.; Eriksson, G.; Gheribi, A. E.; Hack, K.; Jung, I. H.; Kang, Y. B.; Melançon, J.; Pelton, A. D.; Petersen, S.; Robelin, C.; Sangster, J.; Spencer, P.; Van Ende, M. A., FactSage thermochemical software and databases, 2010–2016. *Calphad* **2016,** 54, (Supplement C), 35-53.
